# Population Subdivision of Japanese Flounder *Paralichthys olivaceus* in the Pacific Coast of Tohoku Japan Detected by Means of Mitochondrial Phylogenetic Information

**DOI:** 10.3390/ijms14010954

**Published:** 2013-01-07

**Authors:** Yuya Shigenobu, Michio Yoneda, Yutaka Kurita, Daisuke Ambe, Kenji Saitoh

**Affiliations:** 1 Research Center for Fisheries Oceanography and Marine Ecosystem, National Research Institute of Fisheries Science, Fisheries Research Agency, Fukuura, Kanazawa, Yokohama 236-8648, Japan; E-Mail: ambe@affrc.go.jp; 2 National Research Institute of Fisheries and Environmental Inland Sea Hakatajima Station, Fisheries Research Agency, Kinoura 2780, Hakata, Imabari 794-2305, Japan; E-Mail: myoneda@affrc.go.jp; 3 Coastal Fisheries and Aquaculture Division, Tohoku National Fisheries Research Institute, Fisheries Research Agency, Shinhama, Shiogama 985-0001, Japan; E-Mail: kurita@affrc.go.jp; 4 Research Center for Aquatic Genomics, National Research Institute of Fisheries Science, Fisheries Research Agency, Fukuura, Kanazawa, Yokohama 236-8648, Japan; E-Mail: ksaitoh@affrc.go.jp

**Keywords:** marine fish, Pleuronectiformes, gene flow, haplotype, fixation index, phylogenetic information

## Abstract

This study deals with mitochondrial phylogenetic information of Japanese flounder in the Pacific coast of Tohoku Japan to estimate the genetic population subdivision that was undetectable by conventional population statistics. We determined complete sequences of mitochondrial NADH dehydrogenase subunit-2 (ND2) and subunit-5 (ND5) genes for 151 individuals from northern (Aomori and Iwate prefectures, 40–41°N) and southern (Miyagi and Fukushima prefectures, 37–38°N) waters. Samples from both waters showed high genetic diversity, including 126 haplotypes. These haplotypes were located at mixed and nested positions on an inferred phylogenetic tree, and traditional F-statistics indicated no significant population divergence (ϕ_ST_ = −0.00335, *p* > 0.05), corroborating our previous study. Three variable sites, however, showed significant base composition heterogeneity between samples from the northern and southern waters (Fisher’s exact-test, *p* < 0.01). Nucleotide substitutions at the three sites converged on an apical clade, which consisted of the five southern individuals, whereas its sister clade consisted only of the three northern individuals. This phylogenetic information corroborates previous ecological studies indicating the presence of separate stocks in the northern and southern waters.

## 1. Introduction

Many marine organisms spawn pelagic eggs, and their larvae are capable of dispersal over long distances [1]. Significant gene flow in such species has been found among populations over wide geographic regions [2–8]. These would reflect the assumption that many marine populations operate as genetically open systems [9,10]. Such characteristics of marine organisms would be an impediment to population genetic studies with traditional F-statics based mainly on frequency data [11]. A growing number of studies, however, indicated that pelagic larvae are capable of recruiting back to their source population in marine species [12]. Oceanographic conditions where there are sharp discontinuities of physical and biochemical variables could assist in increasing their tendency to self-recruitment and increasing their vulnerability [13]. Thus, it has been assumed that the relationship between dispersal potential and realized gene flow among marine populations is more complex than previously thought.

Japanese flounder *Paralichthys olivaceus* is widely distributed along the coastal waters off Japan, Korea and China [14,15]. This temperate marine fish inhabiting littoral sandy bottom (shallower than 200 m in depth) broadcasts floating eggs, and the larvae spend pelagic stages for more than a month [16,17]. Ecological and morphological evidence suggested the existence of a subdivision among flounder populations along the coastal waters off Japan [17–24]. These studies are, however, not supported by population genetic works. Population genetic studies detected geographical heterogeneity in some regions, but inferred genetic population structure is inconsistent with ecological and morphological research [25–27]. Elevated genetic diversity within the flounder population and substantial gene flow among stocks [26,27], including ancient admixture between major mitochondrial lineages [27], obscure the population subdivision of the flounder along the Japanese coast.

**Figure 1:**
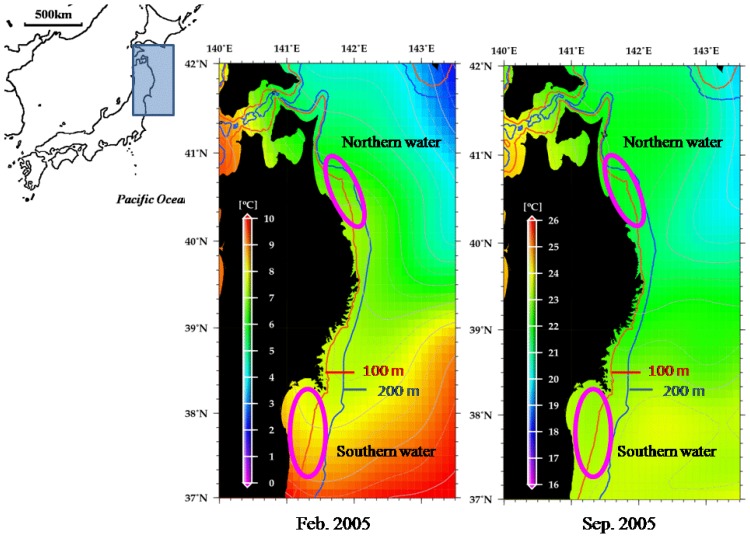
Sampling locations of Japanese flounder used in this study, and sea surface temperatures along the Pacific coast of Tohoku Japan for February and September 2005. The sea surface temperature data set was provided by NCDC, USA [28].

In the Pacific coast off northern Japan, Tohoku district, previous tagging data and life history research suggested two diverged populations, each comprising northern (Iwate and Aomori prefectures, 40–41°N) or southern (Fukushima and Miyagi prefectures, 37–38°N) waters [22–24]. There are limited littoral areas distributed in the intermediate region between the two waters. The oceanographic conditions also differ between the two waters. The northern and southern waters are influenced by Tsushima and Kuroshio warm currents throughout the year, respectively, while in the intermediate region, Oyashio cold current occurs (Figure 1). It would be assumed that such geological and oceanographic conditions in these waters may represent potential barriers to flounder exchange.

The mitochondrial genetic difference between samples from the northern and southern waters, if any, would also be small or undetectable by conventional population statistics, e.g., F_ST_ or even ϕ_ST_ that was suggested to be a better index for population diversification [29]. In an incipient state of population subdivision, haplotypes from phylogenetic clades with various degrees of sequence divergence distribute to each subpopulation in a nested state in which members of clades appear almost evenly in both subpopulations (ϕ_ST_ ≈ 0). However, if migration between subpopulations is limited for a while during which new clades appear by additional nucleotide substitutions, migrants do not carry members of some of these clades by chance. The limited migration thus leaves genetic footprints, *i.e.*, biased distribution of some clades and base composition heterogeneity at sites that define these clades.

In this study, to explore the possibility of genetic divergence of Japanese flounder in the Pacific coast of Tohoku Japan, we tried to reveal genetic footprints of the incipient state of population subdivision with mitochondrial nucleotide variation in relation to the character state along phylogenetic tree.

## 2. Results and Discussion

### 2.1. Genetic Variability of Japanese Flounder

**Figure 2:**
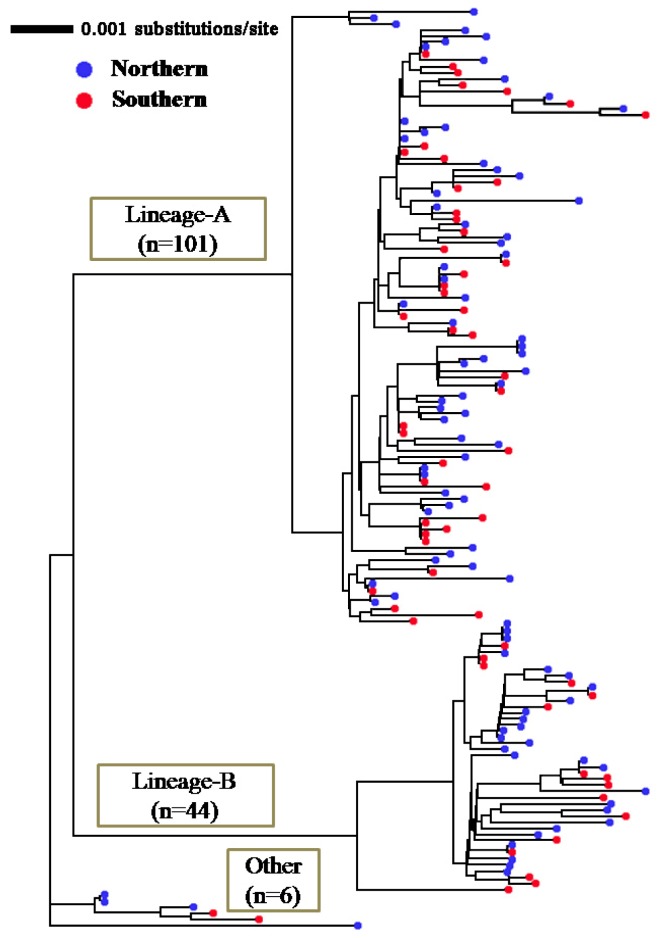
Neighbor-joining tree for 151 individuals of Japanese flounder derived from the concatenated NADH dehydrogenase subunit-2 (ND2) and subunit-5 ND5 sequences. Genetic distances among the individuals were estimated by Tamura-Nei + *I* + *G* distance model.

We determined the whole sequences of NADH dehydrogenase subunit-2 (ND2: 1046 bp) and subunit-5 (ND5: 1839 bp) genes for all 151 individuals. There was no gap in these regions among the material fish examined. The haplotype diversity values (h) of samples from the northern and southern waters were 0.9973 ± 0.0022 and 0.9966 ± 0.0040, respectively. The nucleotide diversity values (π) of the samples from northern and southern waters were 0.0079 ± 0.0039 and 0.0075 ± 0.0037, respectively. The neighbor-joining tree showed the two major mitochondrial clades (lineage-A and -B) with deep branching (Figure 2), as in our previous study [27]. Haplotypes of both mitochondrial lineages coexisted within samples from the northern and southern waters, with several cases of common haplotype between them.

### 2.2. Segregation Sites, Character Mapping and F(ϕ)-Statistics

**Figure 3:**
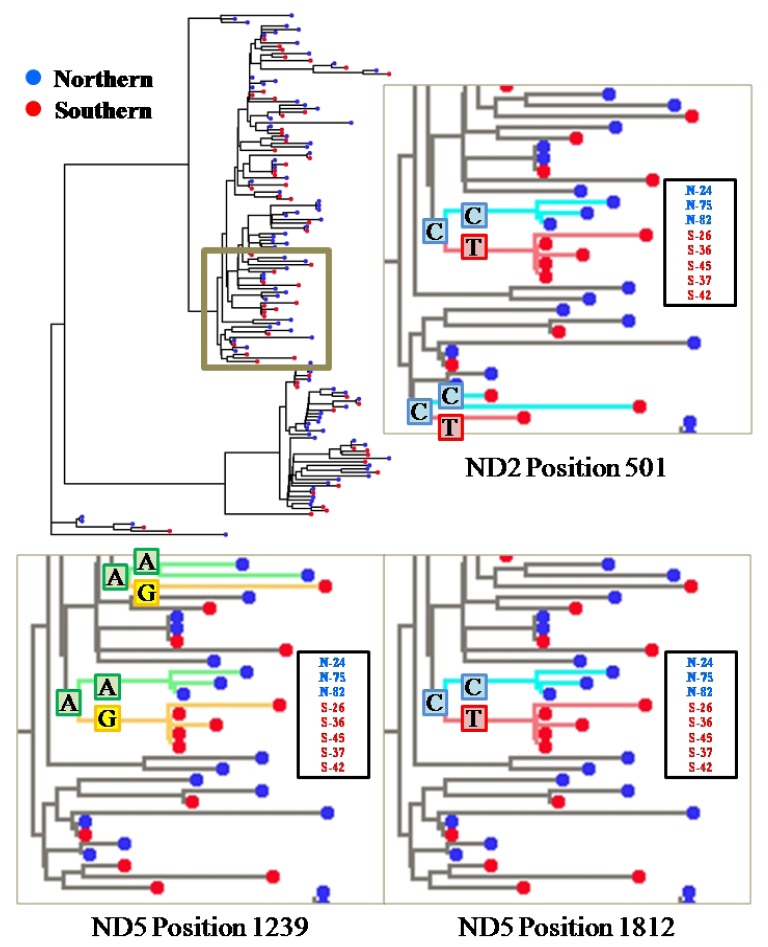
Character assignment for the three synonymous substitution sites (ND2 position 501, ND5 position 1239 and ND5 position 1812) under the accelerated character state optimization. Colored lines indicate the genetic footprint of each variable site. S-26, 36, 37, 42 and 45 are individuals of southern water.

We found three nucleotide sites (ND2 position 501, ND5 position 1239, ND5 position 1812), which show base composition heterogeneity between samples from the northern and southern waters (Fisher’s exact-test, *p* < 0.01) (Table 1). All of these sites were located at third codon positions and contained synonymous transitional base substitutions, whereas other variable sites did not exhibit base composition heterogeneity between samples from the northern and southern waters regardless of being synonymous or nonsynonymous. Character mapping along the phylogenetic tree revealed that the three sites converged on a shallow clade, which consisted of five individuals from the southern water (S-26, S-36, S-37, S-42 and S-45), whereas its sister clade consisted of three from the northern water (N-24, N-75 and N-82) (Figure 3). A “C” to “T” substitution at the ND5 position 1812 was solely mapped at a clade and apomorphic to the five individuals from southern water. The other substitutions (“C” to “T” at the ND2 position 501 and “A” to “G” at the ND5 position 1239) were also mapped at this clade in the same way, though these two substitutions were multiply mapped on other shallow clades separately. On the other hand, to the base composition heterogeneity, there was no significant genetic difference between samples from the northern and southern waters based on the value of fixation index (ϕ_ST_ = −0.00335, *p* > 0.05).

### 2.3. Discussion

Wild Japanese flounder show high mitochondrial haplotype diversity with two major lineages sympatrically distributed along the coast of Japan [27]. Samples from the northern and southern waters of the Pacific coast of Tohoku Japan also show the same genetic property (Figure 2). Flounders harboring these mitochondrial lineages comprise a single panmictic population at each locality, because of Hardy-Weinberg equilibria at microsatellite loci in all the flounder populations from the Japanese coast examined [26]. Co-existence of these deep branched lineages would be of a hybridizing swarm of populations in old geological times. Genetic admixture of previously isolated populations can increase genetic variation [30]. Raised genetic variability, both in haplotype and nucleotide diversity, within population is empirically known to drive F- or related statistics lower [11,29]. Accordingly, such a historical background of Japanese flounder might be a factor of high genetic variability and difficulty in detecting population subdivision.

A value of fixation index (ϕ_ST_ = −0.00335, *p* > 0.05) that is better to display divergence between highly variable populations than F_ST_ [29] suggested that there has been gene flow between the northern and southern waters. Existence of common haplotypes between samples from the northern and southern waters also indicates migration has taken place until recently (mutation has not yet hit the sequenced regions) or even presently.

If the northern and southern waters contain a single panmictic population, every mitochondrial lineage could be detected from both samples evenly. However, we confirmed a sample-specific lineage defined by the three synapomorphic sites (Figure 2). This phylogenetic information corroborates previous ecological studies indicating separate stocks in these waters [18,19,22–24]. Even in the highly connective marine environments, oceanographic fronts could impede admixture among fish populations [13]. Cold water front and littoral bottle-neck between the northern and southern waters indicate that a corridor connecting these two waters for the temperate littoral fish is narrow (Figure 1). Limited migration between the northern and southern waters may thus be responsible for the biased distribution of some clades between the two samples.

Under limited migration between populations, migrants do not or hardly carry haplotypes of some clades by chance. Such limited migration thus leaves genetic footprints on heterogeneity in haplotype distribution and base composition between populations.

Recently, several nuclear DNA markers, which were suggested to be under adaptive evolution, provided valuable results for population genetic studies [31]. Population specific lineages are conspicuous in adaptable environments, even if gene flow among geographic waters is frequent. On the other hand, the three nucleotide substitutions found in the genetic marker employed in this study are at third codon positions and synonymous, and they are most likely neutral or under very week adaptive selection, if any. The present study showed that upon phylogenetic and base composition analyses, even neutral or near neutral genetic markers could indicate small and incipient population subdivision, which is undetectable with conventional population statistics.

## 3. Materials and Methods

### 3.1. Samples

We purchased 91 wild individuals at wholesale fish markets adjunct to fishing ports in Iwate and Aomori Prefectures (northern water, 40–41°N) and 60 in Fukushima and Miyagi Prefectures (southern water, 37–38°N) in 2005 (Figure 1). Because usage of gears for the flounder fishery is regionally regulated, fishermen bring their catch to the nearest fishing port where they belong. We could thus obtain geographically structured samples from these fish markets. We excluded hatchery-reared Japanese flounder, which had abnormal pigmentation [32,33].

### 3.2. Population Genetic Analysis

Extractions of genomic DNAs from muscle tissues followed [34]. We sequenced the mitochondrial ND2 and ND5 genes, using a two-step polymerase chain reaction (PCR) direct sequencing technique. PCR amplification and sequencing strategy followed [27]. The concatenated sequences of ND2 and ND5 genes worked for subsequent analyses.

Calculations of haplotype and nucleotide diversities for each samples followed [35]. We counted the number of variable nucleotide sites by eyes and tested base composition heterogeneity between the two samples at each variable site by Fisher’s exact-test. Phylogenetic relationships among haplotypes were inferred from the neighbor-joining method based on Tamura-Nei + *I* + *G* distance, whose substitution model was selected by AIC comparison, using MEGA5 [36]. Then, the genetic footprints at the variable sites, which show base composition heterogeneity between the two samples, were traced along branches of the neighbor-joining tree by PAUP [37] under the accelerated transition optimization. Fixation index (ϕ_ST_) between the two samples was calculated using Alrequin version 3.11 [38].

## 4. Conclusions

The present study showed that upon phylogenetic and base composition analyses, even neutral or near neutral genetic markers could indicate small and incipient population subdivision, which is undetectable with conventional population statistics. The phylogenetic information of Japanese flounder corroborates previous ecological studies indicating the presence of separate stocks in the northern and southern waters of the Pacific coast of Tohoku Japan.

## Figures and Tables

**Table 1:** The numbers of each nucleotide at the three synonymous substitution sites for which the base composition was statistically different between the northern and southern samples.

	Northern samples				Southern samples				*p*-value of Fisher’s exact-test
	
	A	G	C	T	A	G	C	T	
ND2 Position 501			91				54	6	0.0034
ND5 Position 1239	91				54	6			0.0034
ND5 Position 1812			91				55	5	0.0089
